# Duration mismatch negativity under varying deviant conditions in individuals with high schizotypal traits

**DOI:** 10.3389/fpsyt.2024.1428814

**Published:** 2024-08-06

**Authors:** Jue Deng, Yuanjun Zhang, Liqin Lu, Yuanhua Ou, Xianghui Lai, Siwei Chen, Yiduo Ye

**Affiliations:** ^1^ Cognitive Neuroscience and Abnormal Psychology Laboratory, Department of Penalty Execution, Fujian Police College, Fuzhou, China; ^2^ School of Psychology, Fujian Normal University, Fuzhou, China; ^3^ Department of Forensic Science, Fujian Police College, Fuzhou, China; ^4^ Department of Basic Courses, Fujian Police College, Fuzhou, China

**Keywords:** schizotypy, mismatch negativity (MMN), auditory, duration, schizophrenia

## Abstract

**Background:**

Although impaired auditory mismatch negativity (MMN) has consistently been found in individuals with schizophrenia, there are few and inconsistent reports on nonclinical individuals with schizotypy. To date, no studies have thoroughly assessed MMN with different degrees of deviant oddballs in nonclinical schizotypal samples. The aim of this study was to examine the extent of duration MMN (dMMN) amplitudes under two deviant duration conditions (large and small) in nonclinical participants with high schizotypal traits.

**Methods:**

An extreme-group design was utilized, in which 63 participants from the schizotypy and control groups were selected from a pool of 1519 young adults using the Schizotypal Personality Questionnaire (SPQ). MMN was measured using passive duration oddball paradigms. Basic demographic information and musical backgrounds were assessed and matched, while depression and anxiety were evaluated and controlled for. The repeated measures analysis of covariance was utilized to evaluate differences in dMMN between groups. The Bonferroni correction was applied for multiple comparisons. Partial correlation and multiple linear regression analyses were conducted to investigate the association between dMMN amplitudes and SPQ scores.

**Results:**

The amplitudes of dMMN at Cz were significantly increased under the large deviance condition in nonclinical schizotypal individuals (*F* = 4.36, *p* = .04). Large-deviance dMMN amplitudes at Fz were positively correlated with mild cognitive-perceptual symptoms in the control group (*r_p_* = .42, *p* = .03). However, as schizophrenia-like symptoms worsened and approached the clinical threshold for schizophrenia, small-deviance dMMN amplitudes at Cz showed negative associations with the cognitive-perceptual factor in the schizotypy group (*r_p_* = -.40, *p* = .04).

**Conclusion:**

These results suggest the importance of considering the degree of deviation in duration when implementing the auditory oddball paradigm among nonclinical participants with schizotypal traits. In addition, our findings reveal a potential non-linear relationship between bottom-up auditory processing and the positive dimension of the schizophrenia spectrum.

## Introduction

Schizotypy is a personality construct in the general population characterized by schizophrenia-like symptoms (1–5). It has been conceptualized as an indicator of the latent risk of schizophrenia-spectrum disorders (6–12). Previous behavioral and neurobiological studies have shown that individuals with high schizotypal traits exhibit some schizophrenia-related processing abnormalities and cognitive deficits, although to an attenuated degree (2, 13–15). Schizotypy study allows researchers to investigate schizophrenia-related psychopathology without exposure to the confounding effects of antipsychotic medication (16). Such studies are critical for clarifying various detrimental and protective factors in schizophrenia (5, 17) and enabling early intervention (18).

In terms of identifying vulnerable individuals, distinctions exist between high schizotypy and clinical high risk (CHR), though both can identify individuals at an elevated risk for developing psychosis in community and patient samples (19). Schizotypy refers to the latent susceptibility linked to psychosis and/or schizophrenia proneness, representing the observable manifestation of an underlying vulnerability to traits associated with schizophrenia spectrum disorders. Comparatively, CHR reflects a state vulnerability, denoting a prodromal phase of symptomatic state for psychosis within patient populations (20, 21). This differentiation between trait-based schizotypy assessments and state-based CHR symptoms was corroborated by a longitudinal study indicating significant changes in CHR symptoms while schizotypy traits remained relatively stable over time (22). Hence, screening participants based on measures of schizotypal traits may offer a more accurate representation of the inherent vulnerability in at-risk individuals.

Mismatch negativity (MMN) is an auditory event-related potential (ERP) component evoked by a passive oddball paradigm with deviant auditory stimuli (23, 24). MMN is a sensory memory-based brain response to any discriminable change in a stream of sound stimulation occurring, even in the absence of attention (25, 26). MMN is dominated by bottom-up auditory sensory processes (27), and it provides an objective index for sound-discrimination accuracy (28, 29). Previous studies have reported a relationship between MMN and the functioning of glutamatergic N-methyl-D-aspartate (NMDA) receptors (30–32).

The MMN amplitude is deficient among individuals with schizophrenia (24, 33–35) and is often associated with the severity of schizophrenia-spectrum symptoms (36). Auditory MMN has been commonly regarded as a possible cortical pathophysiological marker of schizophrenia-spectrum disorders (35). Previous studies have proven a robust decrease in MMN amplitude in chronic schizophrenia (37). This reduction is also evident in first-episode schizophrenia, albeit to a lower extent (38, 39). A meta-analysis showed that MMN impairment has a significantly larger effect size in chronic schizophrenia patients than in first-episode schizophrenia (40), indicating that MMN amplitude is associated with disease progression. MMN deficiency is currently an indicator of auditory dysfunction in schizophrenia (41), especially impaired primary auditory processing and poor detection of external acoustic changes (42).

The schizophrenia-spectrum continuum theory (43, 44) proposes that schizotypal traits comprise schizophrenia-like symptoms below the clinical threshold (5). Thus, it seems reasonable to presume that individuals with high schizotypy may exhibit a similar (but mild) reduction in MMN amplitudes and deficiencies in auditory test performance comparable to those exhibited in individuals with schizophrenia. However, reports are few and inconsistent. Baldeweg (45) assessed schizotypal traits and auditory MMN in nonclinical participants and discovered a negative correlation between MMN amplitude and positive factor scores. In contrast, Broyd (46) found that young adults with more positive schizophrenia-like symptoms (suspiciousness) showed larger MMN amplitude. Furthermore, research on first-degree relatives of individuals with schizophrenia-spectrum disorders has reported intact MMN amplitudes in unaffected relatives, who are considered subclinical samples with a genetic risk of schizophrenia (40, 47–49). In addition, larger MMN amplitudes have been reported in individuals with schizotypal personality disorder (50).

The above contradicting reports can be tentatively interpreted in two ways. The first interpretation is related to the diversity of schizotypal individuals. Some studies used the extreme-group design and allocated individuals to discrete groups (based on very low or very high schizotypy scores) or screened high-risk participants with psychotic-like symptoms (45, 47, 51). In contrast, other studies used the non-extreme-group design based on a correlational or median split (46, 52). As such, the degree of schizophrenia-like symptoms varied widely among studies. One meta-analysis reported that in studies on schizotypy, the extreme-group design might reveal more pronounced effects (14).

The second tentative interpretation concerns the level of deviance in oddball sounds. The effects of MMN may be associated with the parameter of deviant stimuli (53). Horton (54) reported that the between-group difference might be most sensitive when the degree of deviation in the odd stimulus is just enough to induce the maximum MMN amplitude in controls. However, auditory sensitivity varies greatly among individuals, and there is no agreement regarding the optimal level of variation in oddball sounds. The parameters that regulate deviancy in the auditory oddball paradigm varied greatly across studies on the schizophrenia spectrum (24, 42, 46, 54–57). Therefore, using sounds with different levels of deviancy in a single study can help clarify schizotypy-related abnormalities in MMN.

This study accounted for the factors described above. Here, we used the extreme-group design to screen participants and examine auditory MMN amplitudes using a duration oddball paradigm with two levels (small and large). Although patients with first-episode schizophrenia and individuals at high clinical risk have been found to exhibit reduced duration and pitch MMN (35), the effect in duration MMN was more robust than the effect in pitch MMN (23, 24).

We implemented a comparison between groups to investigate duration MMN (dMMN) in individuals with high schizotypy. Then, a correlation analysis was performed between dMMN and schizotypy in each group to evaluate whether the association between schizotypy and dMMN is consistent across different deviance levels. Participants with highly schizotypal traits were expected to show distinct dMMN patterns compared with the controls, since previous studies have reported schizophrenia-spectrum-related abnormalities in MMN and auditory function (33, 35, 41, 46). However, owing to previous inconsistent results on MMN amplitude from different samples with varying degrees of schizophrenia-spectrum symptoms (46, 50), we did not propose any specific trend hypotheses in this study. Instead, we probed the possibility of non-linear associations between schizotypal traits and MMN. However, we did hypothesize the presence of distinct patterns of correlation between schizotypy and dMMN evoked by different levels of deviant stimuli. This prediction was based on the inconsistent results across studies that used different parameters when designing the auditory oddball paradigm (23, 24, 42, 54, 56).

## Material and methods

### Participants

#### Procedure of screen and recruitment

Sixty-three participants were selected for the experiment from a pool of 1519 university students.

The selection process involved the quantitative evaluation of schizotypal traits using the schizotypal personality questionnaire (SPQ), either online or offline. After a minimum of one month, participants who scored in the top 15% on a preliminary test were sent an online re-test questionnaire. This re-test included the SPQ as well as a survey regarding the participants’ demographic information and history of mental or hearing-related illnesses. Based on the responses, individuals who met the inclusion criteria were invited to participate in the study. These criteria included having a total SPQ score in the top 15% of the sample on both preliminary test and re-test, not being a musical major, having no hearing impairment or substance dependence, and not having a diagnosis of schizophrenia-spectrum disorders for either the individual or any of their first-degree relatives. Thirty-one individuals with high schizotypy agreed to participate in the study and formed the Schizotypy group (SP).

The online questionnaire was also sent to individuals who scored below average on the SPQ test. From the respondents, we selected the 32 participants who were most closely matched to the Schizotypy group to serve as the control group (CG). The screening process for the control group mirrored that of the Schizotypy group, except for the criterion of below-average SPQ scores on both tests.

Neither SP nor CG participants had a history of head trauma or neurological disorders, and all participants exhibited right-handedness.

#### Measures

The ordinal α of SPQ is 0.74–0.84 among Chinese individuals, and the dimensions include “cognitive-perceptual,” “interpersonal,” and “disorganized,” representing the positive, negative, and disorganized factors of schizophrenia, respectively (58, 59).

The intelligence quotient (IQ) was estimated using the brief Wechsler adult intelligence scale (60). The depression status was assessed with the Beck depression inventory (61), and anxiety was evaluated through the self-rating anxiety scale (62). Parental socioeconomic status (SES), a composite score consisting of three dimensions (parental education, parental occupation, and family property), was also assessed (63). We used Grison’s revised criteria to quantify the participants’ musical background (64, 65) because the MMN effect may be associated with musical training or experience (66).

IQ, depression, and anxiety were assessed when the participants arrived at the laboratory. Other data were collected via the online questionnaire. All participants were asked not to stay up late or consume alcohol 24 hours before the experiment. The local ethics review committee approved this study. The study was performed in full compliance with the Declaration of Helsinki. All participants provided written informed consent before data acquisition and were financially reimbursed. The reimbursement ranged from ¥60 to ¥100 for each participant, depending on their attitude to the experiment, which included factors such as punctuality and completion of all tests.

### MMN paradigm

MMN was obtained via the classical passive duration oddball paradigms. Participants were instructed to ignore sounds and watch a silent movie intently (a nature documentary from the British Broadcasting Corporation). Stimuli were presented binaurally through over-ear headphones (Bose QC35 II). The stimuli sequence consisted of 800 pure sinusoidal tones (1000 Hz), including 656 standard stimuli (82%; 50 ms) and 144 deviant stimuli (18%). The deviant stimuli contained 72 tones with a large variation in duration (150 ms) and 72 tones with a small variation in duration (100 ms). The volume of all tones was consistent across participants, with a 10 ms rise/fall period. The stimulus onset asynchrony (SOA) was 1000 ms, and the test lasted for 800 s. All sounds were played pseudo-randomly to ensure that at least three stimuli at the beginning of the sequence and at least two stimuli between deviated tones were standard.

### Electroencephalography data acquisition and preprocessing

Electroencephalographic (EEG) data were acquired at a sampling rate of 1000 Hz using a 64-channel NeuroScan EEG system (10–20 layout; Neuroscan, Germany) while the participant was seated in an anechoic chamber. Bipolar recordings of horizontal and vertical electrooculogram activity were obtained from the supra-/sub-orbital and external canthi sites. The electrodes were referenced to the nose tip, with the impedances being maintained below 10 kΩ. Electrical activity was recorded with an analog filter bandpass of 0.1–100 Hz and stored on a hard drive for offline analysis.

Data were analyzed offline using EEGLAB (v13.6.5b) and ERPLAB (v7.0.0), which are open-source Matlab (R2013b)-based software programs for EEG analysis. Filters were applied from 0.1 to 30 Hz, with a notch filter at 50 Hz. Eye-blink and eye movement artifacts were corrected through independent component analysis (ICA). Noise were evaluated and rejected via automated routine of ERPLAB (v7.0.0). Following this, epochs were extracted from -100 to 800 ms relative to the stimulus onset. Baseline correction was applied concerning a 100 ms pre-stimulus window. Epochs containing artifacts (exceeding ±75 μV) at each electrode were excluded from the analysis. Data from three participants in CG and three in SP were excluded because more than one-third of their epochs had artifacts. Therefore, data from 29 CG and 28 SP participants were included in subsequent analyses.

ERP waveforms for standard and deviant stimuli were acquired by across-trial averaging. Difference waveforms were calculated by subtracting the standard ERP from deviant ERPs separately for each level of variation (large or small). Considering previous reports on the time window and topography of MMN (46, 67–69), combined with our visual examination of the waveforms in this study, we selected two electrodes (Fz and Cz) for analysis. We computed the amplitudes at 180–300 ms post-stimulus and calculated the mean dMMN amplitude. There was no significant between-group difference in the number of total remaining epochs or the number of remaining epochs under each condition (*p* >.31). In addition, there was no obvious separation in the latency of average waveforms. Therefore, the latency of dMMN was not included in subsequent statistical analyses.

### Statistical analyses

SPSS software (v22.0; IBM Corp., USA) was used for statistical analysis. Differences in demographic data between groups were assessed using an independent sample *t*-test or a chi-square test. We used repeated measures analysis of covariance (ANCOVA), with deviant level (large and small) as the within-subject factor, group as the between-subject factor (SP and CG), the dMMN amplitude as the dependent variable. Given that negative emotions have been shown to potentially affect mismatch negativity (MMN) amplitude (67, 70), in order to control for the possible impact of mood status along with schizotypy, the depression and anxiety scores were included as covariates in the analysis if they differed between groups. The Bonferroni correction was applied for all multiple comparisons.

Partial correlation and multiple linear regression analyses were performed to explore the relationships between dMMN amplitudes and SPQ scores. Depression and anxiety scores were included as covariates in relevant analyses, if they varied between groups. Considering the mixed results of previous studies (33, 35, 41, 45, 46, 50), a quadratic regression analysis would be a tentative method to explore the potential non-linear relationship between dMMN amplitudes and SPQ scores, once the results of the current data showed a latent trend of nonlinearity.

## Results

### Demographic comparisons

The basic information and SPQ scores of participants can be found in **Table 1**. **Table 1** also indicates that anxiety and depression scores were significantly higher in the SP compared to the CG (p <.001). As a result, the scores for anxiety and depression were included as covariates in the subsequent statistical analysis. There were no significant between-group differences in age, gender, parental SES, education, IQ, or musical background.

**Table 1:** Demographic characteristics of the study groups.

	SP			CG			*t/*χ^2^	*p*
	*n*	mean/ percentage	*SD*	*n*	mean/ percentage	*SD*		
Age (years)	31	20.36	2.30	32	20.52	2.45	-0.27	0.79
Gender							0.01	0.92
Male	12	38.7%		12	37.5%			
Female	19	61.3%		20	62.5%			
Parental SES	31	-0.01	0.26	32	0.01	0.32	-0.35	0.73
Education	31			32			0.13	0.72
Undergraduates	28	90.3%		28	87.5%			
Postgraduates	3	9.7%		4	12.5%			
IQ	31	107.23	9.32	32	109.72	7.58	-1.17	0.25
Music background	31	2.77	0.72	32	2.69	0.90	0.42	0.67
SPQ total score	31	51.42	6.06	32	11.22	5.12	28.49	**< 0.001**
Cognitive perceptual	31	22.23	3.55	32	6.17	2.93	19.63	**< 0.001**
Interpersonal	31	16.61	3.01	32	3.09	2.12	20.70	**< 0.001**
Disorganized	31	12.58	1.90	32	1.95	1.63	23.85	**< 0.001**
Anxiety	31	45.81/ 32.3%	8.96	32	35.06/ 3.1%	6.01	5.60	**< 0.001**
Depression	31	7.32/ 61.3%	6.07	32	1.34/ 3.1%	1.43	5.42	**< 0.001**

IQ, intelligence quotient; SES, socioeconomic status; SPQ, schizotypal personality questionnaire; SP, schizotypy group; CG, control group; SD, standard deviation.

The bold value means the between-group difference is significant.

#### Group differences

**Figure 1:**
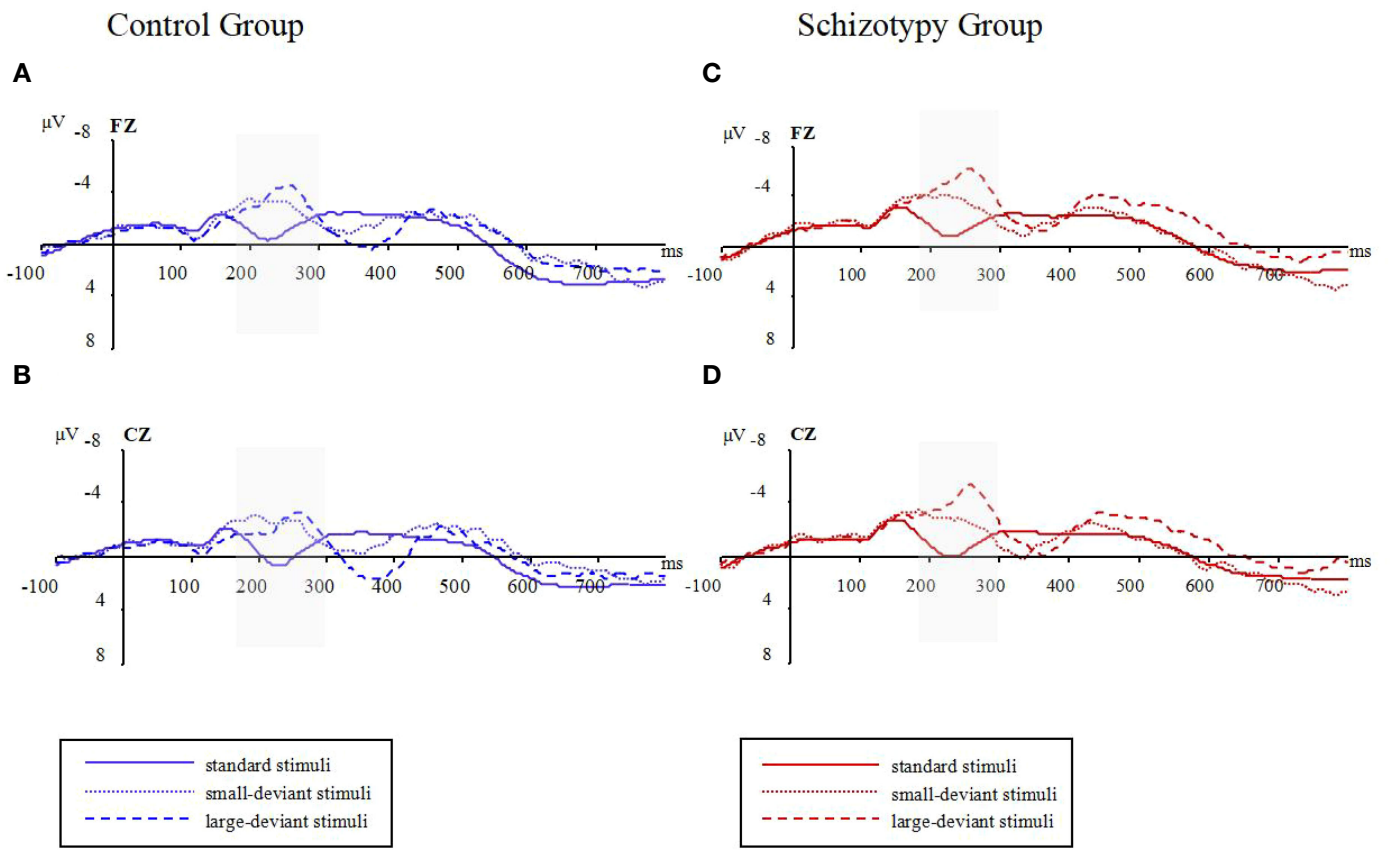
Raw waveforms in three oddball conditions for two groups; **(A)**: waveforms at Fz electrode for the control group; **(B)**: waveforms at Cz electrode for the control group; **(C)**: waveforms at Fz electrode for the schizotypy group; **(D)**: waveforms at Cz electrode for the schizotypy group. Gray-shaded areas represent the focused time-window; ms, milliseconds; μV, microvolts.

The raw waveforms under the conditions with standard stimuli and two types of deviant stimuli can be found in **Figure 1**. The mean amplitudes between 180 and 300 ms post-stimulus were presented in **Table 2**.

**Table 2:** Mean amplitudes between 180 and 300 ms post-stimulus at the Fz and Cz electrodes under three conditions.

	Fz						Cz					
	CG		SP				CG		SP			
	*M*	*SE*	*M*	*SE*	*t*	*p*	*M*	*SE*	*M*	*SE*	*t*	*p*
Standard	-1.01	0.40	-1.46	0.66	0.59	0.56	-0.17	0.32	-0.67	0.51	0.84	0.40
Small-deviant	-2.81	0.57	-3.42	0.68	0.69	0.50	-2.24	0.48	-2.47	0.61	0.28	0.78
Large-deviant	-3.32	0.53	-4.82	0.82	1.55	0.13	-2.18	0.45	-3.85	0.64	2.16	**0.04**

M, mean; SE, standard error; SP, schizotypy group; CG, control group.

The bold value means the between-group difference is significant.

**Figure 2:**
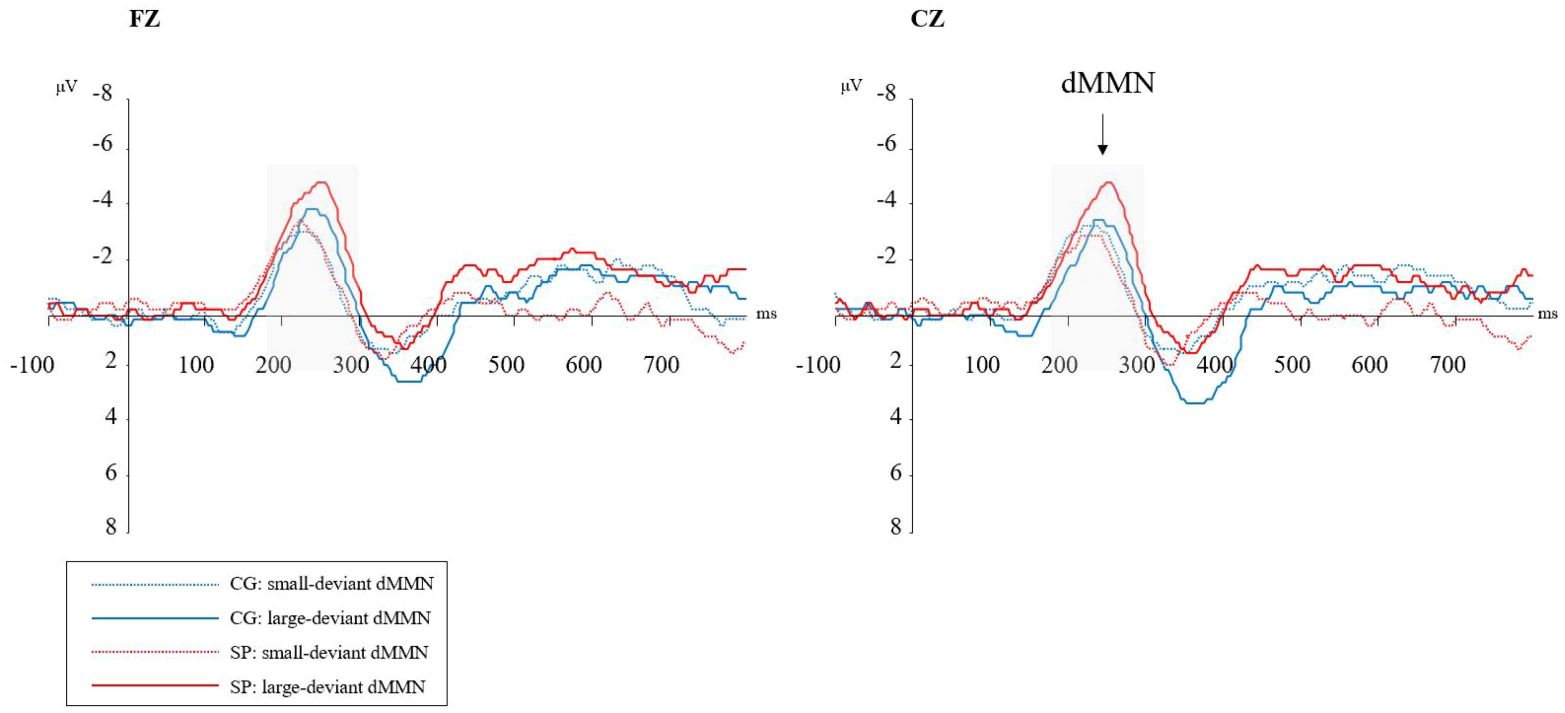
Duration mismatch negativity (dMMN) waveforms at the Fz and Cz electrodes. SP, schizotypy group; CG, control group; ms, milliseconds; μV, microvolts. Gray-shaded areas represent the time-window of dMMN.

**Figure 3:**
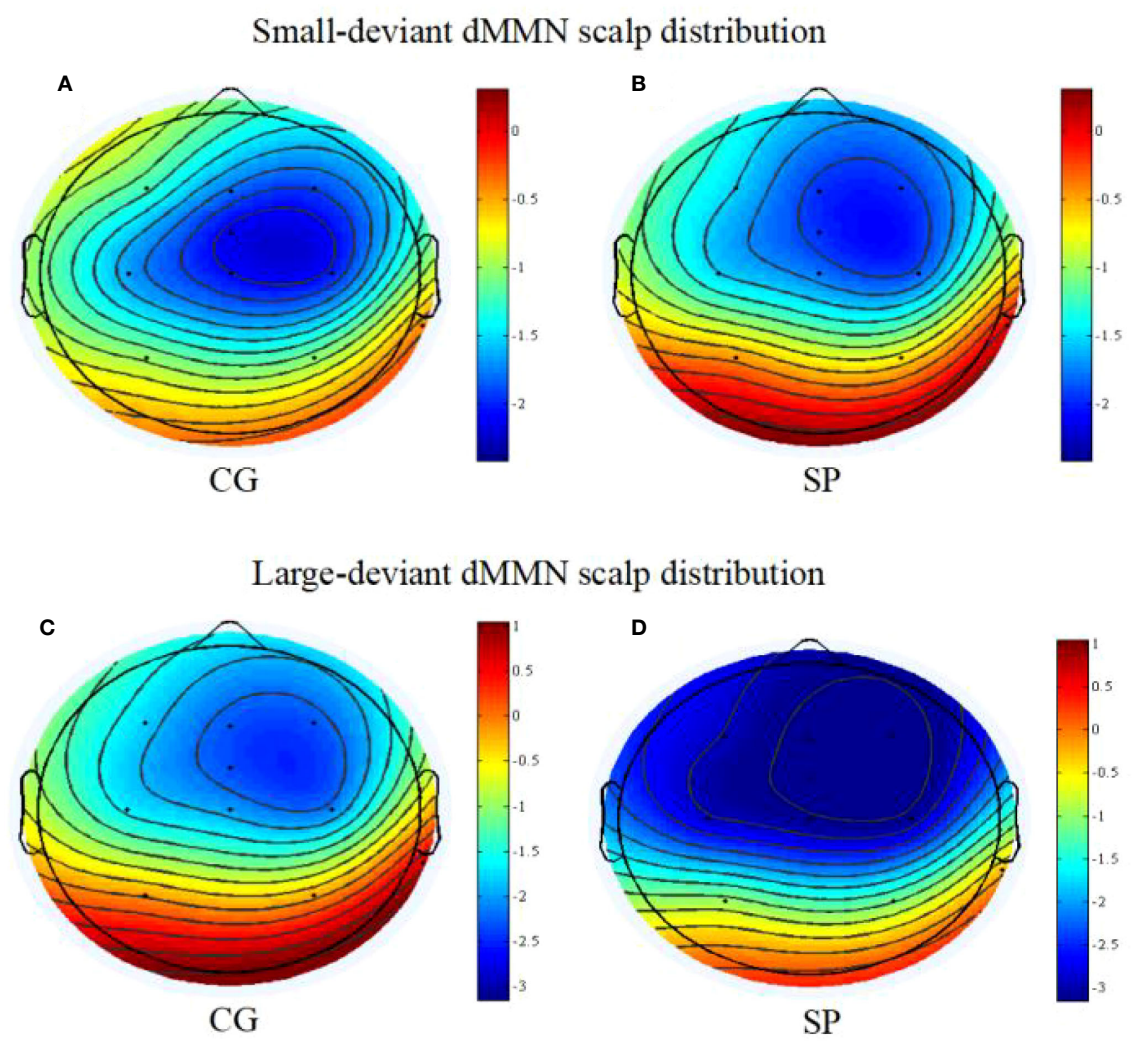
The scalp topography maps of dMMN (between 180 and 300ms post-stimulus); **(A)** the scalp distribution of dMMN under the small deviant oddball condition for the control group; **(B)** the scalp distribution of dMMN under the small deviant oddball condition for the schizotypy group; **(C)** the scalp distribution of dMMN under the large deviant oddball condition for the control group; **(D)** the scalp distribution of dMMN under the large deviant oddball condition for the schizotypy group. SP, schizotypy group; CG, control group.

**Figure 2** shows the waveforms of dMMN from the SP and CG. **Figure 3** shows the scalp distributions of dMMN. At the Fz site (**Figure 2**), a significant interaction effect (*F*(1,53) = 4.27, *p* = .04, η_p_^2^ = .08) was discovered in repeated measures ANCOVA, while there being no significant between-group difference in the large (*F* = 2.56, *p* = .12) or small (*F* = .05, *p* = .82) deviant conditions. There was no significant main effect of the level of duration deviant (*F*(1,53) = 1.09, *p* = .30, η_p_^2^ = .02) or group (*F*(1,53) = .04, *p* = .95, η_p_^2^ <.01).

At the Cz site (**Figure 2**), a significant interaction effect (*F*(1,53) = 5.58, *p* = .02, η_p_^2^ = .10) was discovered. A simple effect analysis revealed that dMMN amplitudes at Cz were significant higher in the SP under the large deviant condition (*M_SP_* = -3.18, *SE_SP_* = .44; *M_CG_* = -2.01, SE*_CG_* = .35; *F* = 4.36, *p* = .04). There was no significant main effect of deviant condition (*F*(1,53) = .51, *p* = .48, η_p_^2^ = .01) or group (*F*(1,53) = .13, *p* = .72, η_p_^2^<.01) in the repeated measures ANCOVA.

#### Relationship between dMMN amplitudes and schizotypy

**Figure 4:**
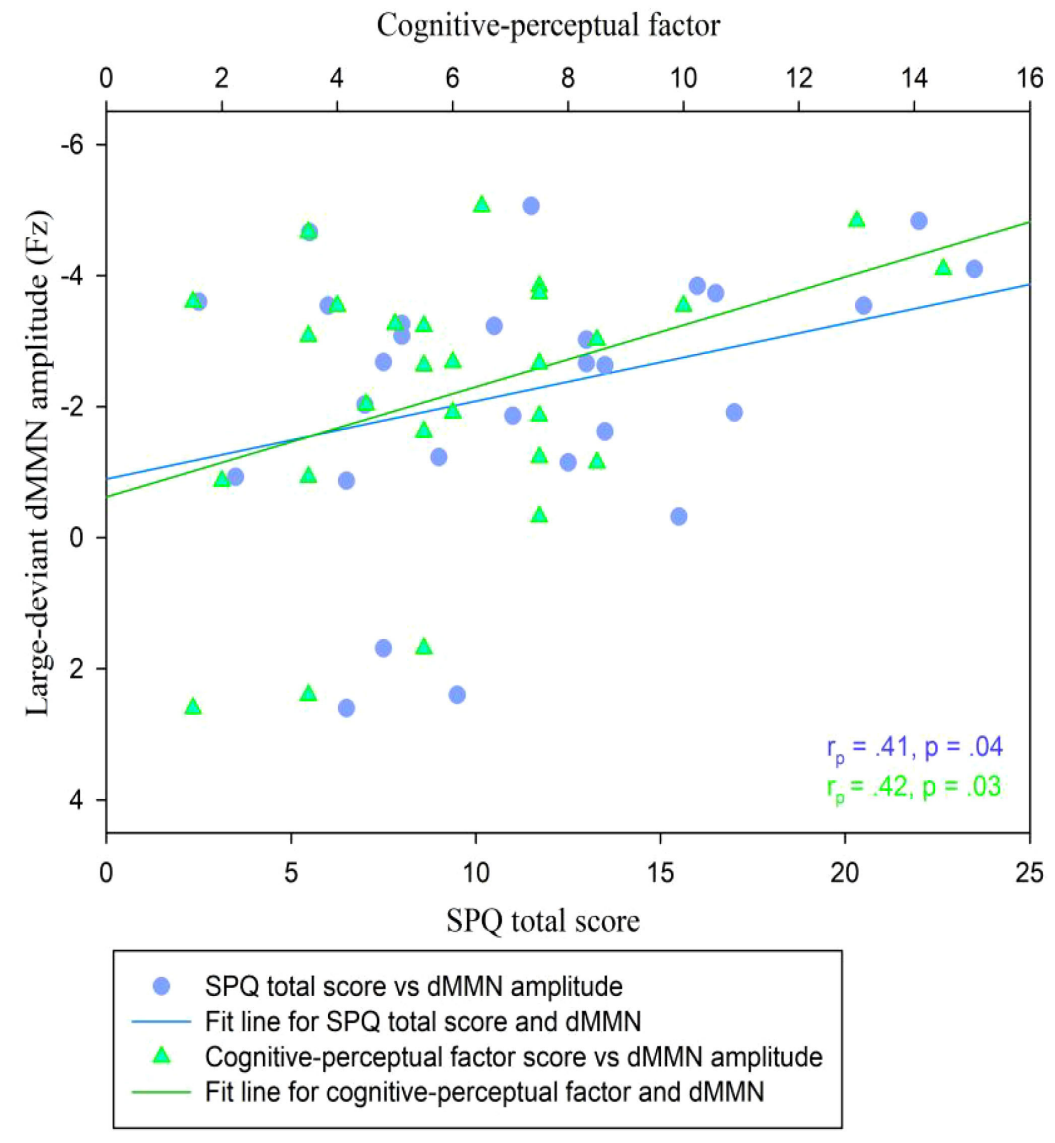
Scatter plot showing linear correlations between dMMN amplitudes at the Fz electrode and SPQ scores (total and cognitive-perceptual dimension) in the CG (n = 29). dMMN, duration mismatch negativity; SPQ, schizotypal personality questionnaire; CG, control group.

In the CG (*n* = 29), the dMMN amplitudes at Fz under the large deviant condition were significantly correlated with SPQ total scores (*r_p_* = .41, *p* = .04; **Table 3**; **Figure 4**) and cognitive-perceptual factor scores (*r_p_* = .42, *p* = .03; **Table 3**; **Figure 4**). Multiple regression analysis in the CG revealed a significant positive predictive effect with SPQ total scores as the predictor, dMMN amplitude at Fz as the outcome variable, and depression and anxiety as covariates (**Table 4**). A similar positive effect was found with the cognitive-perceptual factor as the predictive variable (**Table 4**). However, in the SP group (*n* = 28), the dMMN amplitudes at Fz under large or small deviance conditions were not significantly correlated with SPQ total scores or cognitive-perceptual factor scores (**Table 5**).

**Table 3:** Partial correlations between SPQ and dMMN amplitude, with depression and anxiety scores as covariates, for CG participants (n = 29).

	dMMN amplitude at Fz site		dMMN amplitude at Cz site	
	Small deviant	Large deviant	Small deviant	Large deviant
SPQ	-0.07	0.41*	-0.17	0.36
Cognitive-perceptual	-0.07	0.42*	-0.19	0.37
Interpersonal	0.16	0.28	0.18	0.20
Disorganized	-0.26	0.17	-0.37	0.21

*Correlation is significant at the p <.05 level. SPQ, schizotypal personality questionnaire; dMMN, duration mismatch negativity; CG, control group.

The bold value indicates the correlation is statistically significant.

**Table 4:** Multiple regression analysis of large-deviance dMMN amplitudes at the Fz site concerning SPQ total scores and cognitive-perceptual factor scores, with depression and anxiety included in the model, for CG participants (n = 29).

Outcome variable	Predictor	*B*	*SE*	*Beta*	*t*	*p*	Model properties
dMMN amplitude of large deviance (Fz)	(Constant)	0.66	2.16		0.31	0.76	*R^2^* = 0.26;
	**SPQ scores**	**0.16**	**0.07**	**0.42**	**2.19**	**0.04**	adj *R^2^* = 0.17;
	depression	-0.58	0.27	-0.43	-2.18	0.04	*F* = 2.80;
	anxiety	0.02	0.07	0.05	0.24	0.81	*p* = 0.06
dMMN amplitude of large deviance (Fz)	(Constant)	0.05	2.17		0.02	0.98	*R^2^* = 0.27;
	**Cognitive-perceptual factor scores**	**0.27**	**0.12**	**0.41**	**2.92**	**0.03**	adj *R^2^* = 0.18;
	depression	-0.50	0.26	-0.36	-1.88	0.07	*F* = 2.96;
	anxiety	0.03	0.07	0.10	0.52	0.61	*p* = 0.05

SPQ, schizotypal personality questionnaire; dMMN, duration mismatch negativity; SE, standard error; CG, control group.

The bold value indicates the correlation is statistically significant.

**Table 5:** Partial correlations between SPQ and dMMN amplitude, with depression and anxiety scores as covariates, for SP participants (n = 28).

	dMMN amplitude at Fz site		dMMN amplitude at Cz site	
	Small deviant	Large deviant	Small deviant	Large deviant
SPQ	-0.22	-0.14	-0.24	-0.10
Cognitive-perceptual	-0.37	0.03	-0.40*	0.06
Interpersonal	-0.01	-0.31	-0.02	-0.29
Disorganized	-0.01	-0.02	0.01	0.03

*Correlation is significant at the p <.05 level. SPQ, schizotypal personality questionnaire; dMMN, duration mismatch negativity; SP, schizotypy group.

The bold value indicates the correlation is statistically significant.

**Figure 5:**
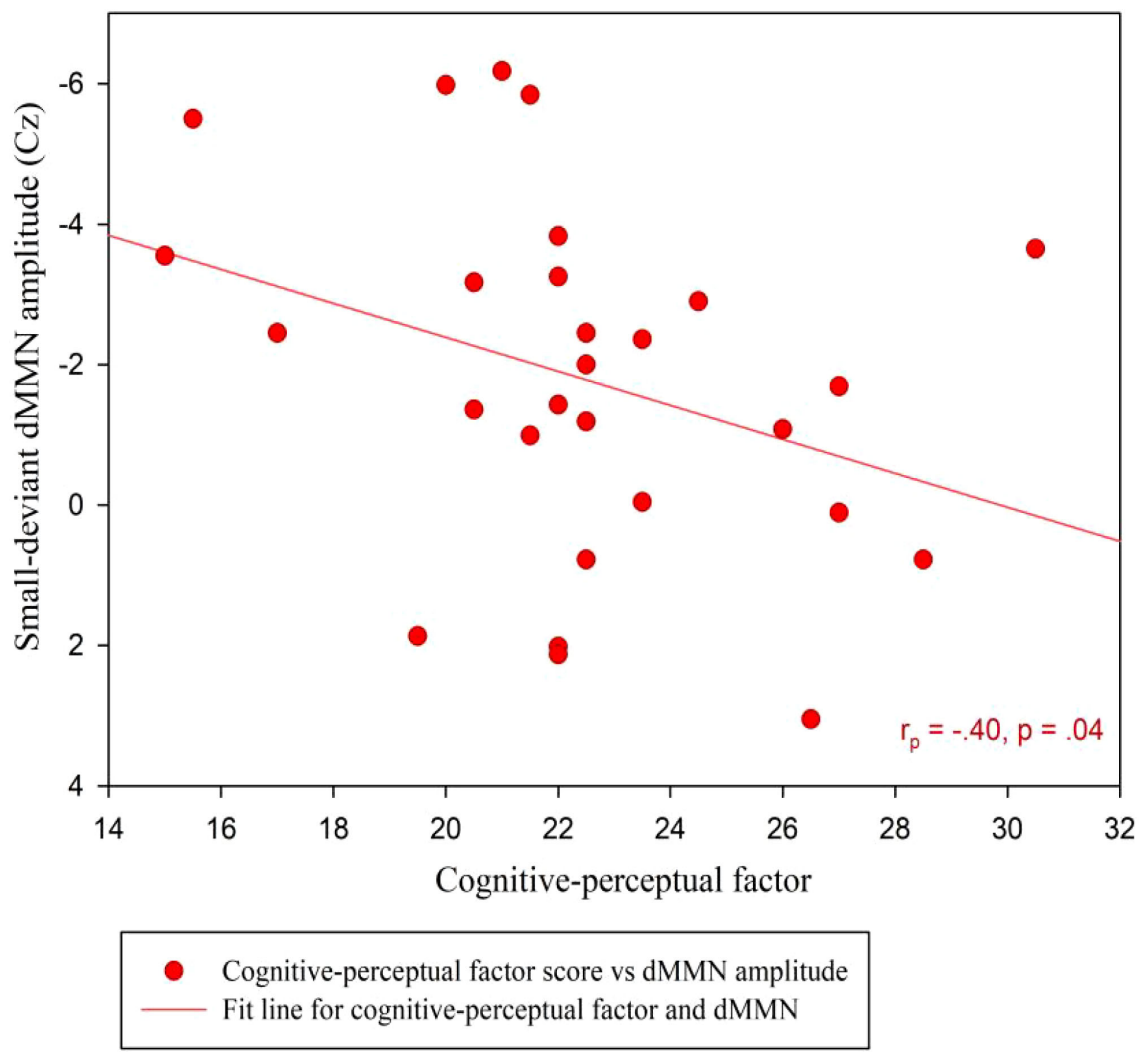
Scatter plot showing linear correlations between dMMN amplitudes at the Cz electrode and the cognitive-perceptual dimension score in the SP (n = 28). dMMN, duration mismatch negativity; SP, schizotypy group.

At the Cz site, there was no significant partial correlation between SPQ total scores or factor scores and dMMN amplitudes under large or small deviance conditions in the CG (**Table 3**). In the SP group, there was a significant negative association between cognitive-perceptual factor scores and dMMN amplitudes under the small deviance condition (*r_p_* = -.40, *p* = .04; **Figure 5**; **Table 5**). Multiple regression analysis in the SP revealed a significant negative predictive effect with cognitive-perceptual factor scores as the predictor, dMMN amplitude at Cz as the outcome variable, and depression and anxiety as covariates (**Table 6**).

**Table 6:** Multiple regression analysis of small-deviance dMMN amplitudes at the Cz site concerning the cognitive-perceptual factor scores, with depression and anxiety included in the model, for SP participants (n = 28).

Outcome variable	Predictor	*B*	*SE*	*Beta*	*t*	*p*	Model properties
dMMN amplitude of small deviance (Cz)	(Constant)	6.53	3.29		1.98	0.06	*R^2^* = 0.26;
	**Cognitive-perceptual factor scores**	**-0.28**	**0.13**	**-0.39**	**-2.16**	**0.04**	adj *R^2^* = 0.16;
	depression	0.14	0.08	0.36	1.81	0.08	*F* = 2.74;
	anxiety	0.01	0.05	0.04	0.21	0.83	*p* = 0.06

dMMN, duration mismatch negativity; SE, standard error; SP, schizotypy group.

The bold value indicates the correlation is statistically significant.

**Figure 6:**
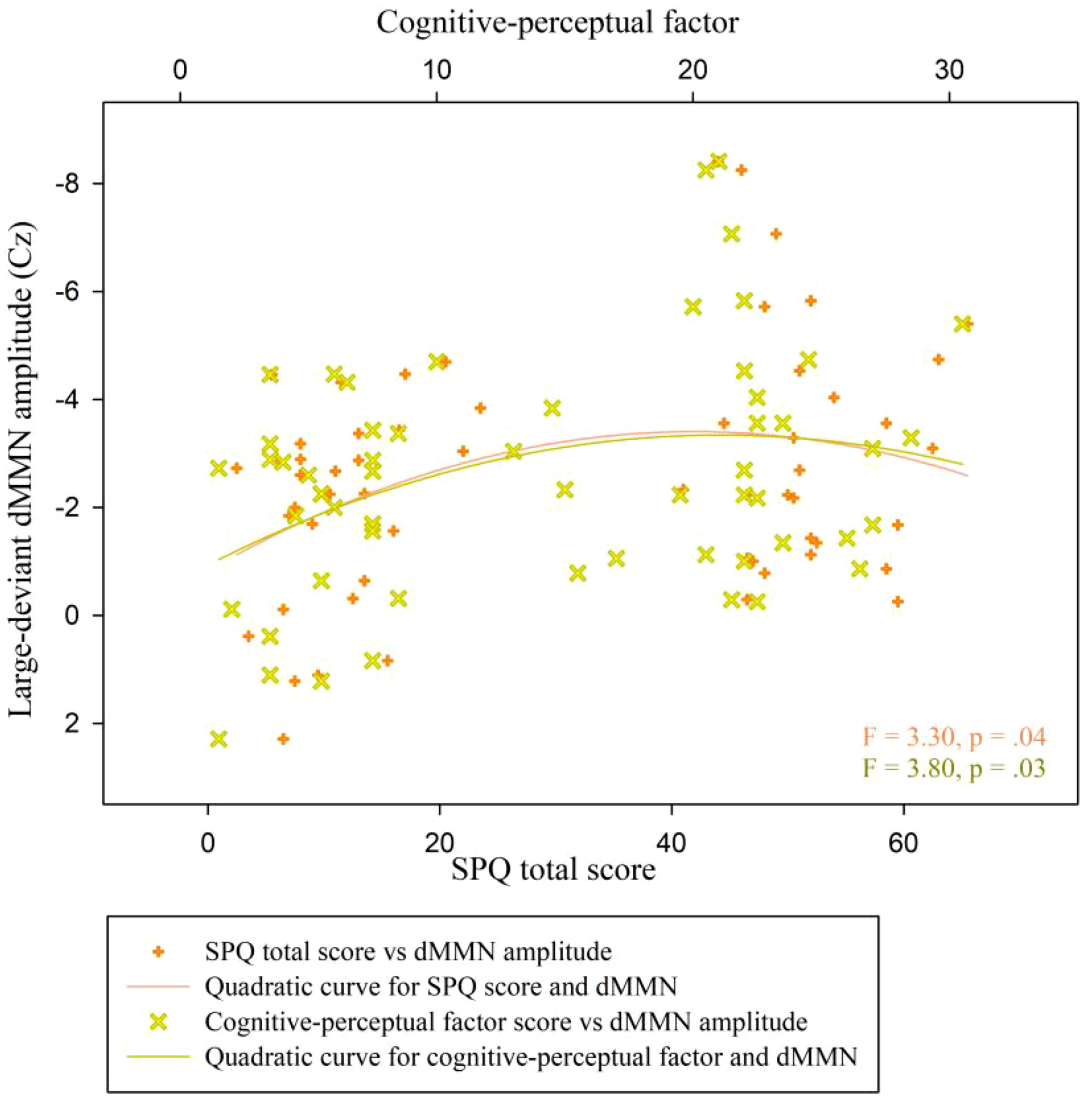
Scatter plot showing a quadratic relationship between dMMN amplitude at the Cz electrode and SPQ scores (total and cognitive-perceptual dimension) across both groups (n = 57). dMMN, duration mismatch negativity; SPQ, schizotypal personality questionnaire; CG, control group.

A quadratic regression analysis was performed to analyze the data of all participants (*n* = 57). We found a significant quadratic relationship between the SPQ total scores and the duration MMN amplitude at Cz under the large deviant condition (y = 0.82 + 0.12x-0.002x^2^; *R^2^* = .11, *F* = 3.30, *p* = .04; **Figure 6**, inflection point [41.0, 3.33]). The analog equation between cognitive-perceptual scores and the duration MMN amplitudes at Cz under the large deviant condition also showed a significant quadratic relationship (y = 0.66 + 0.25x-0.01x^2^; *R^2^* = .12, *F* = 3.80, *p* = .03; **Figure 6**, inflection point [21.17, 3.35]). No quadratic correlation was found between other schizotypal traits and dMMN amplitudes.

## Discussion

To our knowledge, this is the first study to investigate dMMN using different deviant levels of acoustic parameters in nonclinical individuals with high schizotypal traits. Our study yielded the following main findings: Firstly, under the large deviant condition, participants with high schizotypy exhibited significantly higher dMMN amplitudes at the Cz electrode compared to controls; Secondly, in the CG, dMMN amplitudes in response to large-deviance stimuli showed a positive correlation with schizotypal traits, particularly cognitive-perceptual factor scores. Moreover, the relationship between dMMN amplitudes and schizotypal traits, specifically cognitive-perceptual factors, appeared to be non-linear. Thirdly, within SP, small-deviance dMMN amplitudes demonstrated a negative association with cognitive-perceptual factors. These findings partially support our hypotheses and indicate that abnormalities in auditory MMN vary according to changes in the deviant level of auditory stimuli among individuals exhibiting subclinical schizophrenia-like traits. The subsequent sections discuss the sample characteristics and parameters of auditory stimuli used in the oddball paradigm from two distinct perspectives.

### Similarities and differences with schizophrenia

Most clinical studies on auditory MMN and schizophrenia reported a deficiency in MMN amplitudes (33, 35, 36, 71, 72), regardless of the degree of the deviant stimulus. However, we found increased MMN under the large deviant condition among subclinical individuals, which is inconsistent with the findings of most previous clinical studies but consistent with two studies. One focused on the general population (46), and one used clinical samples with schizotypal personality disorder (50). We also found that dMMN amplitudes under the small deviant condition were not significantly different between the SP and CG groups. However, this is also inconsistent with most previous findings but similar to some results based on healthy first-degree relatives of individuals with schizophrenia (40, 47–49). Overall, our results can be interpreted as follows.

First, MMN is likely to be intact among individuals at genetic risk or with mild schizotypal traits below the clinical threshold for schizophrenia. Deficiency in MMN amplitudes may arise as the cognitive decline that accompanies schizophrenia (46, 47) or may be present as a function of a psychotic illness that has already emerged (41). The current results support this viewpoint. Specifically, impaired MMN amplitudes may be an endophenotypic marker for clinical schizophrenic symptoms related to dysfunction of the primary auditory cortex (73) rather than mild or moderate schizophrenia-like traits in the healthy population or individuals at risk of schizophrenia-spectrum disorders (47).

Second, the above explanation does not contradict the notion of the schizophrenia-spectrum continuum (43, 44). Instead, our results indicate that the bottom-up auditory processing of temporal information, particularly the automatic detection of obvious change in sound duration, may show non-linear rather than linear better-to-worse variations related to schizophrenia-spectrum. This interpretation regarding a non-linear correlation was proposed tentatively in a previous study, where the researchers performed auditory threshold tests among participants with highly schizotypal traits (17) and found a significantly higher sensitivity to stimulus duration among SP participants. The positive correlation and quadratic relationship between large-deviance dMMN amplitudes and cognitive-perceptual factor scores support this non-linear interpretation. Overall, these results supported a potential “inverted-U” relationship between these variables, previously shown to describe the association pattern between creativity and psychotic symptoms (74). However, the underlying mechanism is unknown (75).

It should be noted that the negative associations between schizotypy and MMN amplitudes in the SP do not contradict the non-linear explanation. Instead, this result can be incorporated into the “inverted-U” association pattern. In this case, it would indicate the emergent decline of primary auditory processing and aggravated symptoms that are extremely close to the clinical threshold for schizophrenia (SPQ scores higher than the inflection point of the quadratic fit line). This is similar to previous findings in patients with schizophrenia (36, 41, 42). The degree of variation in auditory stimuli likely plays an important role in these relationships, as discussed in section *4.2*.

Third, the two explanations above may indicate potential protection and decompensation processes in the auditory modality according to the connectivity decompensation hypothesis, which is based on the possibility of the gradual neurobiological development of schizophrenia (5, 76). Higher MMN amplitudes reflect enhanced bottom-up auditory processing and improved detection of changes in external sounds (67, 77). As such, they may also function as a protective factor compensating for slight abnormalities related to mild and controllable schizotypal symptoms (5). However, when schizophrenia-like symptoms become aggravated, the protective effects of auditory hypersensitivity may gradually lose efficacy. This can lead to decompensation and descending MMN amplitudes, representing the dysfunction of NMDA receptors and decreased auditory sensitivity (31, 67). SP participants with extremely high SPQ scores in this study were likely in the early stages of this decompensation (5). In addition, auditory hallucination—the most pronounced symptom of schizophrenia-spectrum disorders (78, 79)—may be related to the pathologically dynamic interactions between auditory hypersensitivity and hyposensitivity.

### Level of variation in the oddball stimulus

Although the deviant condition’s main effect was insignificant, the prominent interaction effect between groups and the degree of deviation indicated that variation level in auditory stimuli has an important effect on the correlation between schizotypy and dMMN amplitudes. The pronounced between-group difference was only evident in large-deviance conditions for dMMN amplitudes. Therefore, this shows that increases in MMN amplitudes among SP participants may be limited to obvious variations of external sounds. This explanation supports the positive association between schizotypy and large-deviance (but not small-deviance) dMMN amplitudes.

Recent research suggests that the mismatch negativity (MMN) reflects dynamic cognitive functions at the time of testing, rather than serving as an indication of enduring symptomatology (80). Prior studies have indicated a tendency to overestimate volatility in individuals with schizophrenia (81), with beliefs about environmental variability being linked to increased activity in the dorsolateral prefrontal cortex in those with schizophrenia spectrum disorders (82). This overestimation of external information variability may contribute to a positive correlation between dMMN amplitudes and cognitive-perceptual symptoms, potentially explaining why some patients initially exhibit hyperfunctional auditory processing in the acute phase (e.g., hypersensitivity, auditory hallucinations), followed by chronic negative symptoms (e.g., social withdrawal, affective flattening), which could serve as a protective mechanism against overload. Further research is required to delve into the underlying mechanisms, particularly exploring the association between variability overestimation and MMN in relation to positive and negative symptoms separately.

When the deviation was subtle, there was no significant between-group difference in dMMN amplitudes. However, in the SP group, the dMMN amplitude was negatively correlated with schizotypy under the small-deviance condition. This is consistent with previous results based on clinical samples (24, 30, 33–36, 71). These results verified the impaired auditory MMN effect related to schizophrenia-spectrum symptoms, but only under the small-deviance condition. Schizophrenia-related auditory impairments may begin with deficiencies in automatically detecting subtle changes in external sounds; with schizophrenia exacerbation, symptoms gradually aggravate so that obvious changes in external sounds cannot be efficaciously detected.

In addition, dMMN amplitudes were associated with only cognitive-perceptual factor scores but the interpersonal or disorganized factor scores. It indicated that the bottom-up processing of sound duration might be correlated with the positive dimension of the schizophrenia-spectrum rather than the negative or disorganized dimension. Hence, this supports the view that impairment-related neuro-mechanisms differ between the positive and negative symptoms of schizophrenia (18).

### Limitations

This study had some limitations. First, the degree of sound deviation affected dMMN in individuals with highly schizotypal traits, however, we found no significant main effect of deviant level condition. Second, future studies should test and examine other levels and types of deviation (such as various deviations in pitch or intensity). Third, across-group quadratic correlations between large-deviance dMMN and schizotypal traits should be verified in more continuous samples. The MMN effect in individuals with schizotypy is expected to be investigated using a more comprehensive and optimal experimental paradigm.

## Conclusion

In summary, this study demonstrates different schizotypy-related abnormalities in auditory MMN under diverse deviation levels in duration among nonclinical individuals. The results extend previous findings using various levels of oddball stimuli. We elucidated how variations in duration stimuli affect MMN amplitudes among individuals with highly schizotypal traits. We highlight the necessity of considering change parameters in the duration when implementing the auditory oddball paradigm with schizophrenia-spectrum-related participants. In addition, we found an intact duration MMN effect among nonclinical individuals with high schizotypal traits, indicating that this ERP component may not be an endophenotypic marker for moderate schizophrenia-like traits or psychotic risk in a healthy population. Finally, there may be a non-linear association between the sensitivity of automatic auditory detection and positive schizotypal traits. A trend of quadratic correlation between dMMN amplitudes and cognitive-perceptual factor scores manifested this.

## Data availability statement

The raw data supporting the conclusions of this article will be made available by the authors, without undue reservation.

## Ethics statement

The studies involving humans were approved by Ethics Committee of School of Psychology, Fujian Normal University. The studies were conducted in accordance with the local legislation and institutional requirements. The participants provided their written informed consent to participate in this study.

## Author contributions

JD: Conceptualization, Formal analysis, Methodology, Writing – original draft, Writing – review & editing, Data curation, Funding acquisition, Investigation, Resources, Validation, Visualization. YZ: Data curation, Formal analysis, Investigation, Methodology, Writing – original draft. LL: Formal analysis, Software, Visualization, Writing – review & editing. YO: Conceptualization, Formal analysis, Funding acquisition, Methodology, Writing – review & editing. XL: Data curation, Formal analysis, Investigation, Resources, Writing – review & editing. SC: Conceptualization, Formal analysis, Investigation, Methodology, Software, Writing – original draft. YY: Conceptualization, Funding acquisition, Project administration, Resources, Supervision, Writing – review & editing.
